# Delivery of Oligonucleotides: Efficiency with Lipid Conjugation and Clinical Outcome

**DOI:** 10.3390/pharmaceutics14020342

**Published:** 2022-02-01

**Authors:** Phuc Tran, Tsigereda Weldemichael, Zhichao Liu, Hong-yu Li

**Affiliations:** 1 Department of Pharmaceutical Sciences, College of Pharmacy, University of Arkansas for Medical Sciences, Little Rock, AR 72205, USA; PDTran@uams.edu (P.T.); TGWeldemichael@uams.edu (T.W.); 2 Division of Bioinformatics and Biostatistics, National Center for Toxicological Research, US Food and Drug Administration, Jefferson, AR 72079, USA; zhichao.liu@fda.hhs.gov

**Keywords:** oligonucleotide, lipid conjugates, LNP, delivery, cholesterol, fatty acid, tocopherol, squalene

## Abstract

Oligonucleotides have shifted drug discovery into a new paradigm due to their ability to silence the genes and inhibit protein translation. Importantly, they can drug the un-druggable targets from the conventional small-molecule perspective. Unfortunately, poor cellular permeability and susceptibility to nuclease degradation remain as major hurdles for the development of oligonucleotide therapeutic agents. Studies of safe and effective delivery technique with lipid bioconjugates gains attention to resolve these issues. Our review article summarizes the physicochemical effect of well-studied hydrophobic moieties to enhance the cellular entry of oligonucleotides. The structural impacts of fatty acids, cholesterol, tocopherol, and squalene on cellular internalization and membrane penetration in vitro and in vivo were discussed first. The crucial assays for delivery evaluation within this section were analyzed sequentially. Next, we provided a few successful examples of lipid-conjugated oligonucleotides advanced into clinical studies for treating patients with different medical backgrounds. Finally, we pinpointed current limitations and outlooks in this research field along with opportunities to explore new modifications and efficacy studies.

## 1. Introduction

### 1.1. Background of Oligonucleotide

**Figure 1:**
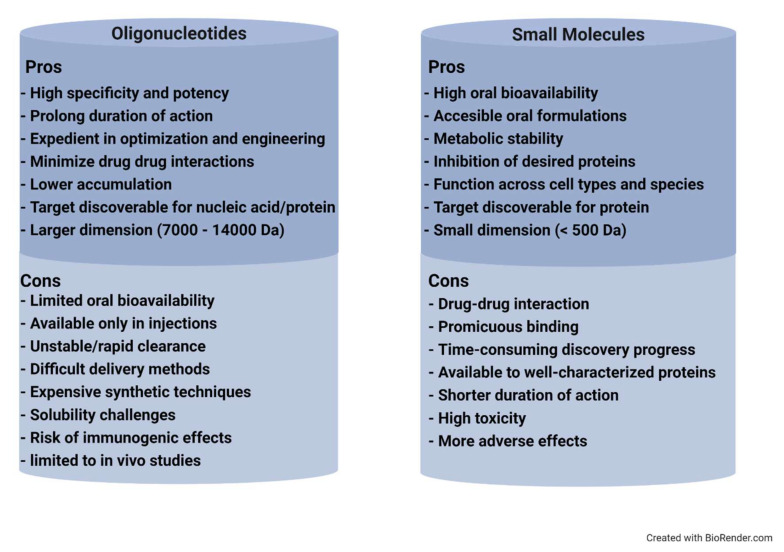
Pros and cons comparison of oligonucleotide versus small molecule drugs. Some highlighted advantages of oligonucleotide would be inhibitory specificity, expedient manufacture, and low in drug–drug interaction. Disadvantages of cost, stability, and formulation remained.

Oligonucleotide (ON) is a short strand of nucleic acid polymers mostly comprising of thirteen to twenty-five nucleotides, which can hybridize to targeted DNA or RNA. They are categorized into classes including antisense oligonucleotides (ASOs), small interfering RNA (siRNA), microRNA (miRNAs), and aptamer. Watson–Crick base pairing is quintessential for ON mechanisms to act on targeted mRNA, which leads to the following consequences: (1) RNase H activity degradation, (2) inhibiting the formation of matured mRNA, and (3) conjuring steric blockage from ribosome interaction [1]. Therefore, ONs are preferable therapeutic strategies to prevent and treat various disorders via selective inhibition of deleterious gene expression. It is indeed shifting the era of drug discoveries into an exciting new field—oligonucleotide therapy. Comparatively, the ease of manufacturing, base-pairing specificity/sensitivity potential, and its long duration of action give higher preference than the conventional therapy. Longer duration of action which varies from weeks to months of post-administration outweighs the technical hitches of being only in an injectable formulation. Given the knowledge of genes and their role accessibility, incurable genetic disorders are made possible through this novel approach. Its application is not merely limited to drug discovery but also pertinent for investigations of the mechanism and stereochemistry of biochemical reactions, mapping of nucleic acid-protein interactions, and diagnostic applications [2]. ON therapy is well aligned to play a noteworthy role in speeding up drug discovery against traditionally undruggable targets. Figure 1 displays some pros and cons of oligonucleotide-based drugs versus conventional small molecules. Hence, ON has gained its deserved attention in research in a wide range of disease indications from oncology to anti-viral therapy. However, the biggest concern for ON is cellular membrane penetration. This hurdle is the result of ON’s highly hydrophilic nucleoside combing of anionic phosphate backbone. Thus, passive transport is not an effective option, and conquering this issue is not a straightforward task.

The assistance with external delivery systems such as liposomes, nanoparticles, or micelles were proposed and experimented thoroughly; however, toxicity was frequently reported due to the immunogenicity caused by polycationic material [3,4]. Alternatively, chemical conjugation to neutral lipid/hydrophobic moiety can overcome this backlash. Hypothetically, these naturally occurring biomolecules that are familiarized with the human system can reduce the risk of toxicity while enhancing cellular penetration and systemic stability. Different forms of lipid structures were incorporated and evaluated in vivo for improvement in pharmacokinetic and pharmacodynamic properties. In this review article, we will summarize the studies of characterizing hydrophobic moiety in the relationship of improving delivery efficacy. Additional discussion about potential therapeutic application and future outcomes of lipid-conjugated oligonucleotides will be highlighted.

### 1.2. Oligonucleotide-Based Drug Mechanism of Action

**Figure 2:**
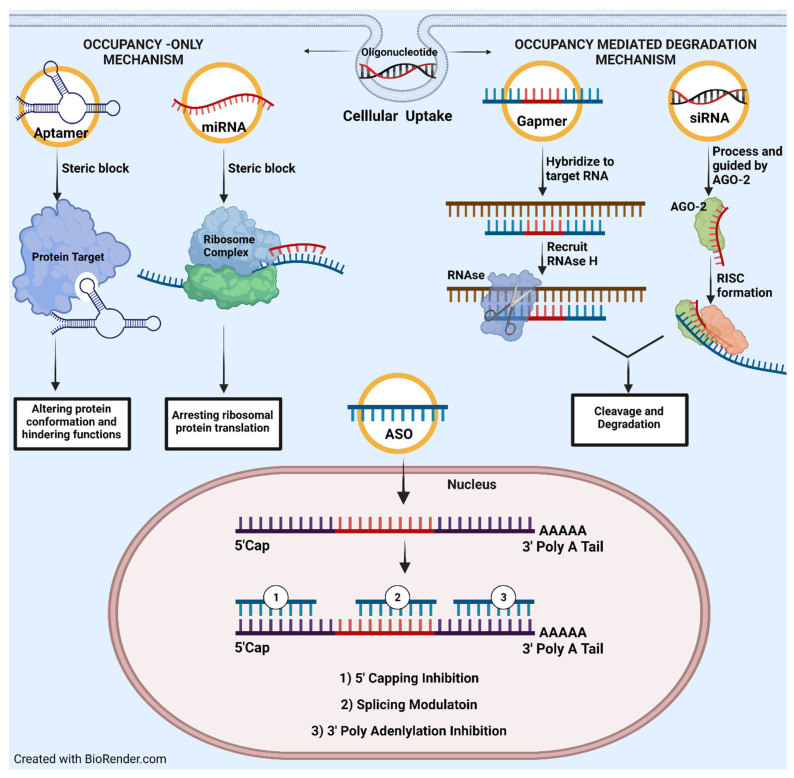
An illustration of main oligonucleotide mechanism of action as divided into two main groups: (1) Occupancy-only and (2) occupancy-mediated degradation. In occupancy only, oligonucleotide would act as steric blocker or inhibitor preventing any upcoming interaction with precedent targets. Meanwhile, occupancy-mediate degradation activates cleavage the targeted RNA via RNase or AGO-2.

A comprehensive review composed by Crooke et al. has highlighted the fundamental aspects of ON mechanism of action. We would like to briefly summarize his work by discussing in two distinctive groups. Occupancy-only mechanism and occupancy-mediated degradation as illustrated in Figure 2. 

In the occupancy-only approach or direct inhibition mechanism, ON will bind specifically to mRNA molecules via Watson–Crick base-pairing, which induces steric block from any followed-up interaction with proteins, nucleic acids, or transcription factors. In consequence, it can conjure either downregulation (translational arrest or cap inhibition) or upregulation (splicing modulation or RNA activation) processes. The most utilized, well studied, and therapeutically beneficial approach would be splicing inhibition. Once targeted mRNA hybridized with ON, a complex of small nuclear ribonucleoprotein (snRNP) would be sterically blocked from intron binding, thus halting the maturation of mRNA [2]. Splicing inhibition was successfully applied for Duchenne muscular dystrophy treatment, for which eteplirsen was approved by the FDA. Another plausible mechanism would be the disruption of RNA structural integrity following by ON hybridization. As result, abnormality in three-dimensional conformation interrupted its stability and halted sequential protein expression. Vickers et al. on disruption of HIV’s TAR element would be a prime example. This research group implemented an ON to destabilize TAR’s (trans-activating response sequence) stem loops, which followed by tarnishing HIV replication efficacy [5].

On the other hand, occupancy-mediated degradation was often emphasized with two major mechanisms: RNase or AGO-2 mediated degradation. Both mechanisms can result in deteriorating targeted mRNA but with slight differences in recruitment algorithms. For RNase, it is universally well documented as an enzyme responsible for degrading a single RNA strand, or RNA:DNA hybrid [6]. It has two isoforms (H1 and H2) which are identified in mammalian cells with expression in the cytoplasm and especially in the nucleus. RNase H1 participates more enthusiastically in cleavage than H2, though H2 is more abundant [7]. Recruitment of RNase is accompanied by a gapmer or even a short tetramer ON. Additionally, binding with an RNA metabolic protein such as P32 can enhance cleavage specificity which provided a good glimpse for optimizing chimera to accelerate RNase H1 activity. AGO-2 protein is one of the four argonaute family members, which facilitates endonuclease cleavage at the targeted RNAs and contains three domains. Mid and PIWI domains confer catalytic actions and perform simultaneously with Paz domain, which is responsible for small RNA binding. Being an indispensable component of the RISC, it operates with highly complementary at the translational or posttranscriptional level. Thus, it exerts RNA-based silencing mechanisms by altering protein synthesis and affecting RNA stability. Precise complementarity between guide RNA and the target is essential for the efficient cleavage of targets. Studies show that mismatches at the 5′ regions are less tolerated than the 3′region of guide RNA or cleavage site [8,9].

### 1.3. Biological Barriers That Challenged Druggability/Pharmacokinetic Profile of Oligonucleotide In Vivo

Dated back in 1998, oligonucleotide was an ultimate breakthrough in the discovery of a new drug modality. Significantly, Fomivirsen [10], a cytomegalovirus (CMV) retinitis ON-based treatment for AIDS immunocompromised patients, was recognized and approved by the FDA. Thus, marked the beginning of its massive emergence in the drug industry. Accounting in 2021, additional ON-based therapies were introduced into the market for non-cancerous indications as shown in Table 1; while there are still numerous entries examined in clinical trials for oncogenic targets [11]. Before approaching this height, the first unmodified ON was deemed expendable due to its high clearance rate after in vivo delivery. Agrawal et al. conducted the first report on the ON pharmacokinetic profile that showed unfavorable properties of unmodified ON after intravenous injection to a monkey with a dose of 30 mg/kg [12]. Quantification of polyacrylamide gel (PAGE) determined short systemic retention of approximately 15 min with a half-life of only 5 min. Structurally, the unmodified ON was identical with the endogenous mRNA in nature. Its highly polyanionic and hydrophilic characters still hampered the ability to penetrate the phospholipid membrane with the addition of high renal clearance.

**Figure 3:**
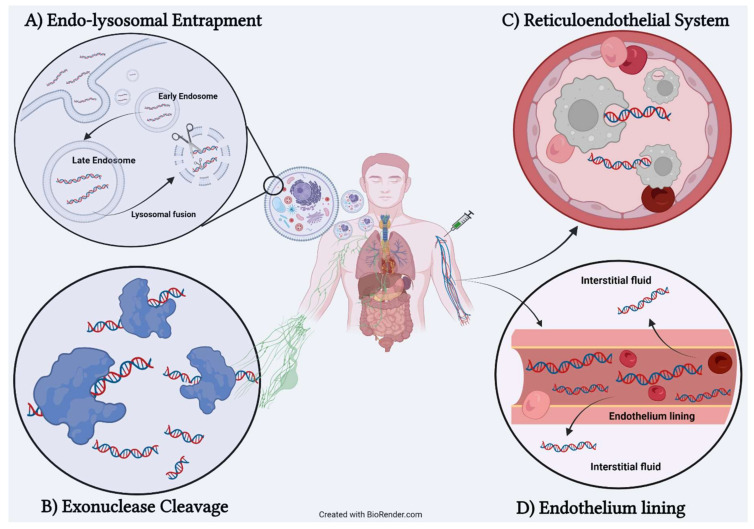
Four biological barriers preventing activity of therapeutic oligonucleotide. (**A**) Endo-lysosomal entrapment. ON required escape from late endosome before subjected to lysosomal degradation. (**B**) Exonuclease cleavage. Initiate hydrolysis of phosphodiester backbone, which deteriorate ON. (**C**) Reticuloendothelial system. Digestion of ON by macrophage can terminate its activity. (**D**) Endothelium lining. Blockage of transverse ON from vascular lumen to interstitial fluid.

From numerous pharmacokinetic data and mechanistic studies, scientists such as Juliano et al. [13] outlined four possible biological barriers that instigate unfavorable conditions for the efficacy of ON therapies as illustrated in Figure 3. We begin the discussion with the first barrier known as nuclease, especially 3′-exonuclease. It is an enzyme that is widely expressed in plasma and induces hydrolyzing reaction by cleaving phosphodiester bond at either at 3′ or 5′ ends. It can directly target ON indiscriminately and catalyze degradation reaction, which leads to complete loss of the therapeutic effect of ON before reaching the targeted site. The second barrier would be the reticuloendothelial system (RES) or mononuclear phagocyte system (MPS). It can easily be defined as a homogenous collection of phagocytotic cells that act as cellular securities to process and clear any form of alienated particles such as toxins, bacteria, or any xenobiotic. Therapeutic ONs are no exception as macrophages engulf ON, which endangers its survivability. Once these phagocytotic cells fused with the lysosome, therapeutic ON are considered dead. Consequently, long-term degradation of ON can lead to detrimental effects such as renal tube degradation, splenomegaly, and elevation of liver transaminase [14]. The third barrier is the thickness of endothelial tissue. The lining of endothelium must be sturdy to enclose safe blood flow; however, in the case of therapeutic ON (which is only administered via injection), the wall of the endothelium can prevent leakage of ON macromolecules. Thus, most therapeutic ON still lingers within circulation while an infinitesimal amount can escape from vascular lumen to interstitial fluid. The final barrier would be the cellular uptake mechanism in which scientists continued to manipulate for ON delivery. They studied the key concept of internalization mechanisms such as clathrin-based coated, caveolar, clathrin-independent carriers (CLIC), or micropinocytosis. As crucial as understanding the uptake mechanism, we beg a question: “How come the cellular uptake can be posed a challenge?” The answer lies in the late endosomal stage after ON is encapsulated in the cytoplasm. Late endosomes are usually fused with the lysosome to break down debris or recycle necessary material; hence, therapeutic ONs are victims of degradation and required to escape for maintaining a longer lifespan [15].

As these hurdles tamper ON effectiveness, alternative solution such as direct nucleic acid modification was applied to overcome exonuclease cleavage. However, it was not adequate since these nucleic acid derivatives were continuously recognized by the immune systems to digest and excrete as foreign invaders. Nanoformulation and direct conjugation (with GalNac, lipid, or antibodies) were strongly recommended to mask ON and avoid from RES associating clearance. Additionally, both techniques could improve membrane penetration, which assist ON to permeate through endothelial lining. Enhancement of ON to escape late endosome-lysosomal degradation remained controversial and not understood clearly. Co-administration with chemical enhancers to disrupt encapsulating vesicle was suggested; however, it concurred with high risk of toxicity. Our Table 1 of FDA approved ON drugs also updated with the modificative modalities to achieve maximal clinical outcome and to bypass the mentioned challenges. 

### 1.4. Early Attempt of Oligonucleotide Chemical Modification 

Direct chemical modification was conceptualized to battle the discussed biological barrier to safely deliver therapeutic ON to its site of action. The earliest attempt was sulfurization of phosphodiester backbone into phosphorothioate (PS), which altered its physiochemical properties. With the low electronegative element of sulfur, phosphorothioate would be less susceptible to be nucleophilic attacked by nuclease. Improvement from the first-generation phosphorothioate was documented with pharmacokinetic data showing extension of half-life up to 1 or 2 h. Moreover, the clearance rate was significantly decreased with less than 5% ON detected in urine or feces after murine dosing for 12 h [16,17]. This high systemic retention was accompanied by a high affinity to plasma protein with 95% bound. Even with phenomenal achievement, there is some evidence of relevant phosphorothioate potential flaws: (1) degradation still can occur via other mechanisms such as transesterification or pyrophosphatase [18] and (2) an excessive amount of phosphorothioate on ON can negatively impact the binding affinity of targeted RNA. This first-generation modification is still frequently applied in modern ON synthesis, but it is incorporated with the second-generation modification at 2′ribose.

For RNA, the 2′ hydroxyl group is a critical component for RNase to recognize and catalyze hydrolysis. Thus, protection of this group is necessary, which can perform via methoxylation. Moreover, 2′ ribose modification was reported to diminish unwanted immune stimulation [19]. Researchers explored this protection technique by starting with naturally occurring 2′-O-methyl (2′-OMe) RNA, which exudes the improvement of nuclease resistance and binding affinity. A bulkier group such as 2′-O-methoxylethyl (2′-MOE) emerged as the most prominent candidate, which can be confirmed via thermal shift assay revealing stronger binding affinity as ΔTm increased from 0.9 °C to 1.7 °C per modification counts. From these encouraging findings, the third generation was developed by introducing a constraint that hindered the nucleotide’s conformational flexibility. The first attempt was bridging 2′-oxygen to 4′-carbon ribose to produce locked nucleic acid (LNA), which showed intense elevation of binding affinity (increasing of ΔTm from 4 °C to 8 °C per modification units binding to RNA) [20]. Its derivatives with an additional methyl group, constrained ethyl (cET), were believed to conduct tighter binding. However, 2′ ribose modification caused therapeutic ON the incompetency to recruit and facilitate RNase cleavage mechanism due to inability to identify and covalently bind to 2′-hydroxyl group [21,22]. A clever solution to this drawback is implementing these second-generation to flank at both sides of the gapmer, an ON consisting of a central DNA region recruiting RNase H.

**Figure 4:**
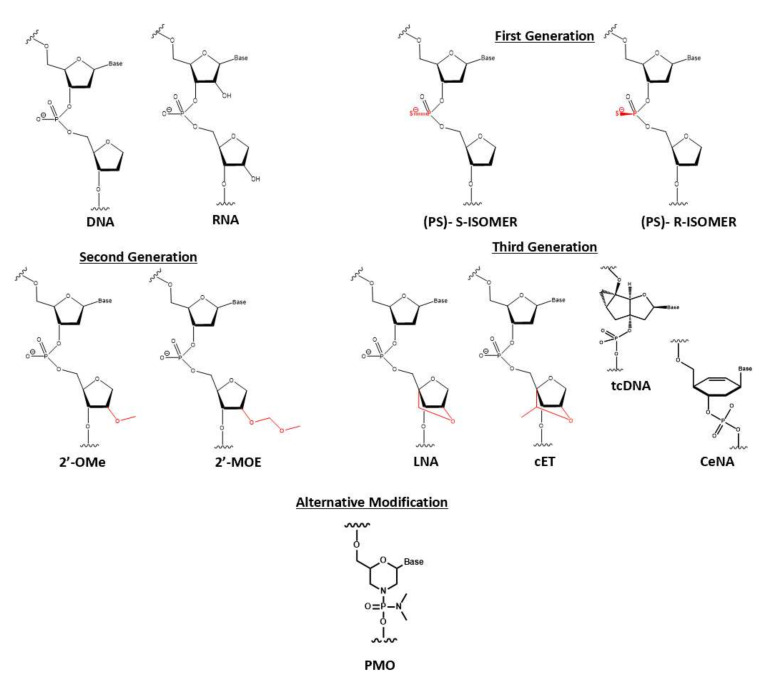
Three generations of common nucleic acid modifications. First generation replaced phosphodiester (PO) backbone to phosphorothioate (PS) to enhance degradative resistance. Second generation includes modification of 2′ ribose into 2′-O-methyl (2′-OMe) and 2′-O-Methoxyethyl (2′-MOE), which are popular in gapmer synthesis. Third generation restricts conformational flexibility by introducing a methyl bridge between 2′-O and 4′ of ribose. Locked nucleic acid (LNA) and constraint ethyl (cET). Ribose moiety would be completely replaced with tricyclic DNA (tcDNA) or cyclohexene (CeDNA). Alternative modification was phosphorodiamidate morpholino oligomer (PMO). This neutral nucleic acid was described with an additional amine accompanied with phosphorodiamidate backbone.

Moreover, ribose moiety can be completely substituted with less rotatable structures such as tricycle DNA (tcDNA) or cyclohexene nucleic acid (CeNA). A fully modified tcDNA, which is equipped with three extra carbons between C(5′) and C(3′), lifted the thermal stability up by 1.2 °C and 2.4 °C per modification while interacting with DNA and RNA, respectively [23]. Similarly, the replacement of a furanose ring with cyclohexene also restricts some flexibility while exhibiting superior serum stability from degradation and enhancing RNase recruitment capability [24]. Nonconventional nucleic acid modification is illustrated via phosphorodiamidate morpholino oligomer (PMO). The ribose moiety retains the traditional oxidative oxygen while being re-closed with an additional ammonia unit. The phosphodiester backbone is replaced with phosphorodiamidate linkage. This modification demonstrates exceptional degradative resistance either from protease, esterase, or 13 different hydrolases in serum and plasma. With the uncharged character, PMO prevents unwanted hybridization with surrounding protein, which exacerbated ON effectiveness [25]. Figure 4 illustrates the representative variation of ON modification segregated by their generation.

Despite these exciting discoveries, systemic toxicity inherited by nucleic acid modification plaque ON therapies as reported in vivo and significantly, clinical trials. For instance, P.S modification was known for enhancing protein plasma binding, however, excessive repetition of P.S in a single ON unit impacted the affinity to mRNA and promiscuously developed off-target toxicity after long-termed exposure [11,21,26]. The second-generation such as 2′-MOE, are encountered in vivo toxicity in mice reported by Zanardi et al. However, there were no significant increases in toxicity for longer treatment duration. cET was the candidate believed to reduce much toxicity compared to other 2′ ribose modifications [27]. Finally, the third generation cannot escape this trauma such as report in LNA with associating liver toxicity. Therefore, the modification must be considered with moderation to avoid unwanted adverse effects and needed additional sources of delivery.

### 1.5. Lipid-Conjugated Oligonucleotides: Method of Delivery and Example of Conjugation

#### 1.5.1. Method of Enhanced Delivery and Lipid-Conjugated Structure

Finding the most optimal and efficient delivery method for therapeutic ON is still an ongoing campaign for the goal of achieving the maximal clinical outcome. Scientists usually implement one of the two following popular approaches: (1) external delivery capsules by utilizing nanoparticles and (2) covalent conjugation of endogenous biomolecules. Naturally occurring substances are preferable with some exceptions for artificial materials. Among these, hydrophobic or lipid moieties have gained much-wanted attention. It is abundant in biological systems and carries out essential functions such as executing signaling transportation. More importantly, it is a body of phospholipid bilayer that can help ON to mimic the hydrophobic properties.

**Figure 5:**
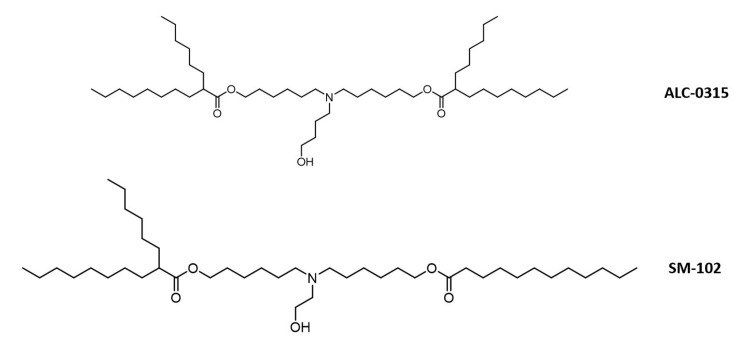
Chemical structure of two most advanced LNP that assisted in delivery of 2 mRNA COVID vaccines. ALC-0315 and SM-102 contains similar structure with ethanolamine head (one is shorter than others). ALC-0315 has two branched degradable ester tail, while SM-102 only has one branched ester tail.

The first exciting investigation of utilizing lipid nanoparticles as ON delivery was conducted by Felgner et al. He incorporated plasmid DNA with cationic lipid such as 1,2-O-octade-anyl-3-trimethylammonium propane (DOTMA) and dioleyl phosphatidyl ethanolamine (DOPE) to induce in vivo transfection into cells. This successful discovery leads to the use of LNP to be drug delivery carriers for small molecules as well. [28]. Significantly, there are eight LNP structures approved by the FDA. Patisiran is an example of ON carried by LNP approved in the market for the treatment of hereditary transthyretin amyloidosis. As by 2021, the most advanced LNP formulation was applied for delivery of two mRNA vaccines, BNT162b2/Comirnaty (Pfizer-BioNTech) and mRNA-1273 (Moderna) to counteract the global SARS-CoV-2 pandemic. For formulation of Comirnaty, ALC-0315 was the main component of this nanoformulation recipe. It was a synthetic lipid-like substance that has an ethanolamine headgroup along with two biodegradable branched ester tails. The LNP was formulated via mixture of ALC-0315/cholesterol/DSPC (Distearoylphosphatidylcholine)/PEG-lipid. In term of mRNA-1273, synthetic lipid SM-102 was selected as primary nanoparticles components. Its structure was similar to ALC-0315 containing an ethanolamine headgroup with difference of one mono and one branched degradable ester tails. mRNA-1273 was formulated strictly with SM-102/DSPC/cholesterol. Both synthetic lipids illustrated in Figure 5 were hypothesized to obtain cone-shaped structure from the branching tails, which boosted the strength of endosomal escape for mRNA molecules [29]. As advancing to clinical trials, Comirnaty was fully approved for individuals 16 years and older while mRNA-1273 is still in the third trial (approved for emergency use). They both encodes for stabilized full-length spike protein but their mRNA contents (100 μg and 30 μg for mRNA-1273 and BNT162b2, respectively). A review by Schoenmaker outlined the abridged and detailed information regarding of dosing and LNP components for both vaccines [30]. However, some ionizable lipids were feared to produce unwanted toxicity and in need of continuously monitoring due to the uncontrolled activation of cytokines after systemic administration [31].

Alternatively, the second approach by lipid conjugation is plausible. Scientific evidence suggests a unity of lipid conjugated oligonucleotide (LON) can reduce the risk of immunogenesis while can maintain tolerance with a high dose in vivo [32]. Similar to LNP, LON’s hydrophobicity is enhanced and be more accessible to membrane permeability and higher rate of internalization. In contrast, LONs are relatively smaller than LNP, which contributes to a higher leakage rate from endothelial lining and perfuse to various tissue types. However, the majority of lipid derivatives are highly accumulated in primary excretory organs, liver and kidney. Administration route is a crucial concept when mentioning LON delivery because it dictates the targeted tissue. In vivo experiments, either intravenous or subcutaneous injections can result in LON migration to clearance organ and some miscellaneous (spleen, skeletal muscle, etc.); while intrathecal or direct cranial injection can occupy parts of the brain [33,34]. Therefore, optimizing delivery location is theoretically a balancing act of hydrophobicity adjustment and understanding the chemical nature of bioconjugates.

**Figure 6:**
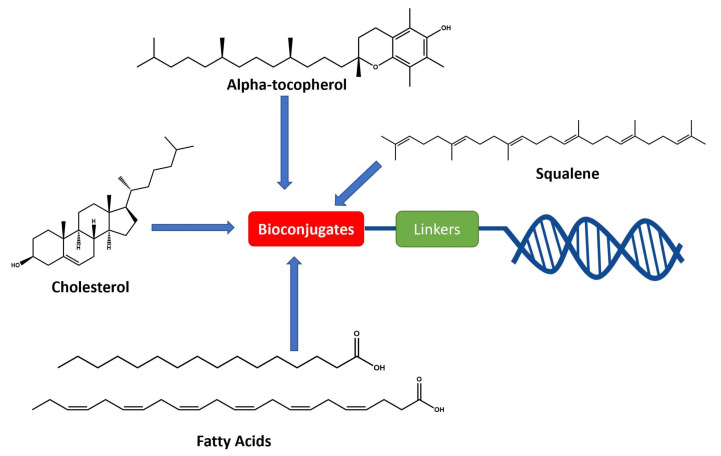
General molecular structure of conjugated oligonucleotides including: (1) synthetic oligonucleotide, (2) linkers, and (3) bioconjugates.

To achieve such a feat, an effective synthesis of LON is required. A fully functional LON consists of three distinct fragments as illustrated in Figure 6: (1) the designed ON (ssRNA, siRNA, aptamer, or any forms), (2) attaching linker, and (3) lipid derivatives. Synthetic LON was produced via either pre-synthetic or post-synthetic approach, which lipoid conjugate would be introduced in a different fashion. An articulate description of both LON’s synthetic routes was reviewed by Raouane and Li et al. [2,35]. We would like to briefly compare two approaches and highlight vital conjugating procedures for lipoid species. When tackling LON with pre-synthetic approach, it provides more flexible lipid point of attachment options. Conjugation can occur either at 3′, 5′ or even between consecutive nucleotides. The most popular and convenient technique is attaching the hydrophilic group at the 5′ end as presynthesized phosphoramidite. However, 3′-lipoid attachment can be arduous because bioconjugation is required to be pre-tethered onto a solid support. For instance, Setsinger premade cholesteroyl solid support via oxidative phosphoramidation [36], while Nikan et al. built a solid support with a pre-formed amide bond with docosahexaenoic acid [37]. Some other example [38] using an alcoholic moiety attaching to a solid support via succinyl linker while bearing another hydroxyl group for cholesterol to attach. Ueno [39] selected glycerol to bridge lipophilic group and mRNA. Interchain bioconjugation was attempted through examples of Guzaev and Durand [40,41]. In contrast, the post-synthetic approach requires two completely independent entities of ON and lipoid conjugation with their complementary reactive group for coupling. Some available techniques could be the formation of triazole linkers resulting from click chemistry reaction between dibenzocycloctyne (DBCO) and azido-lipid conjugates [42]. Raoulane demonstrated the effectiveness of thiol-malamide bridge of RNA and squalene [43].

#### 1.5.2. Example of Conjugations

##### A. Cholesterol Conjugates

Covalently attached cholesteryl moiety as non-vehicle delivery for oligonucleotides was conceptualized as early as the late 1990s [44]. Manoharan’s group claimed 3′- cholesterol-conjugated ON produced the best silencing effect and continued in unfolding the delivery mechanism. They observed a 2-fold uptake increase in comparison to naked ON for silencing murine ICAM-1 and proposed liver uptake mechanism associated with scavenger receptors [45]. Later in the 2000s, scientists from Alnylam synthesized and examined a plethora of cholesteryl derivatives at two terminals of either sense or antisense strands [46,47], which confirmed better in vivo efficacy of 3′-cholesterol ON (3′-Chol ON). Wolfrum et al. expanded Manoharan’s mechanistic notion and elucidated receptor-mediated endocytosis as a key for ON uptake. The cholesterol-conjugated ON was highly recognized and attached to either LDL (low-density lipoprotein) or HDL (high-density lipoprotein); thus, the resulting complex docked to scavenger receptors (SR-BI) and proceed to internalization [48]. At the same time, cholesterol conjugates enhanced the hydrophobicity of oligonucleotide, which ameliorated the drug-like properties. For researching a more feasible pharmacokinetic characterization technique, Godinho et al. attested 3′-Chol ON with rapid distribution phase (*t*_1/2 α_ = 18–33 min) and slow elimination phase (*t*_1/2 β_ = 8–14 h) [49].

A complete pharmacokinetic parameter was summarized in Table 2 for intravenous administration. Nevertheless, it was noticeable that silencing capability can only reach near 50% efficacy even at a low dosage. It was believed that conjugated ON was sequestered during late endosome, which coiled the term endosomal entrapment [50]. Hence, chemical enhancers were used to damage vesicles along the endosomal system to promote escape [51,52]. Additionally, the delivery scope of cholesterol conjugated oligonucleotide was very limited to hepatic cells and to some extend pancreatic cells [53].

Most studies have demonstrated that cholesteryl conjugated ON was delivered effectively, and specifically to liver tissue. Hence, hepatic-related disorder would be its ideal target. For an instant, hypercholesterolemia is the excessive circulation of cholesterol in blood, which caused by either habitual diet or genetic condition. Antisense technology has provided a therapeutic platform through silencing of PCSK9 [54,55,56] or hepatic ApoB [57,58,59,60]; however, the unconjugated ASO treatments suffered mild to serious toxicity while giving questionable efficacy. Henceforth, studies from Wada and Nakajima demonstrated coupling ASO with cholesterol would enhance liver uptake while improving the degradation efficacy of PCSK9 (−50% silencing and 2 µmol/kg) [61] and ApoB (−85% silencing and 0.5 mg/kg) [62], respectively. Both research groups also pinpointed the cleavage of phosphodiester linkage as quintessential for liberating ASO, which showed a 3- to 5-fold increase in vivo potency [63]. Furthermore, the application of cholesterol conjugated ON is extended into the realm of cancer treatment. Liu et al. cholesterol-conjugated let-7a miRNA mimics could downregulate both transcriptional and translational levels of RAS in vitro, and minimize murine xenograft tumor in vivo [64]. Chernolovskaya group delved into silencing MDR1 (multidrug resistance protein), which overexpressed in oncogenic cells to efflux impending drug and amplify resistance [65,66]. 21-mer MDR1 targeting siRNA both as monomer and trimer (63-mer) were compared in vivo demonstrating monomeric siRNA obtained superior silencing efficacy while trimeric derivative accumulated highly in tumors [67,68]. Additional examples of other applicable disease would be including Huntington’s disease [37,69,70], diabetes nephropathy [71], and herpes simplex virus-2 protection [72].

Some cholesterol conjugated ONs were successfully introduced to clinical trials. For example, ARC-520-HBV was the first RNA interference therapeutic for treatment against hepatitis B virus (HBV). ARC-520 injection consisted of a pair of synthetic cholesterol- conjugated siRNAs to augment its delivery to hepatocytes. It also contained polymer-based excipients such as dynamic polyconjugates that enable endosomal escape [73]. Mechanistically, it reduced all RNA transcripts from covalently closed circular DNA (cccDNA) leading to the diminishing of both viral antigen and DNA. ARC-520 was at phase II clinical studies with the promising pharmacokinetic profile in a single-dose study in healthy volunteers. The clinicians found out that IV injection with a dose of 3 mg/kg can increase the curative effect and reduce the viral antigen level by 81–96%. The dosage regimen was given 2 mg/kg once every 4 weeks for 3 total doses. As results, the degree of viral decline and duration of the effect was consistent with the previous animal experiments. Unfortunately, the inclusion of hepatocyte-targeted excipient ARC-EX1 melittin-derived peptide linked to *N*-acetylgalactosamine caused detrimental toxicity in nonhuman primates which rendered the trial to be terminated [74]. In another application, ARO-AAT (SEQUOIA) is currently in phase II/III of clinical trial for the treatment of Alpha-1 antitrypsin deficiency (ATTD)-associated liver disease. The subcutaneous dose of iRNA selectively degraded ATT mRNA caused by Pi*Z mutation. The trial was aimed to determine the safety, tolerability, and pharmacodynamic effect of the drug by gauging the level of plasma and intrahepatic Z-AAT levels. iRNA were given in incremental multiple doses and up to 300 mg in a single shot. It was well-tolerated and resulted in more than 91% serum reduction which was sustained for 6 weeks. To understand dosage window in practical term, this iRNA therapy could be administered four time a year or less to maintain desired silencing effect. The clinical trial is anticipated to be completed by July 2022 (ARO-AAT2001; NCT03945292).

Apart from this, the cholesterol conjugated ON is broadly used for connective tissue growth factor (CTGF) to battle against fibrotic disorders. For instance, Hwang et al. reported a novel application of this modified 2′-OMe phosphorothioate nucleic acid for antifibrotic skin therapeutics. The drug is composed of a cell-penetrating asymmetric interfering RNA (cp-asiRNA) known by OLX10010 as shown in Table 3 (cholesterol conjugate). This iRNA targets the expression of CTGF, and it is currently examined in an ongoing phase 2a clinical trial [75]. When compared to unconjugated siRNA, 1 mmol/L of cp-asiCTGF achieved more than 85% silencing knockdown of CTGF at mRNA level without the assistance of transfection media. The calculated IC_50_ was 0.315 nM in cell lines (the best efficiency was observed in keloid fibroblast cell). Hwang et al. also discussed in vivo studies on rat skin to demonstrate a significant gene-specific silencing capability with 1 mg intradermal injection of lipid modified siRNA after 72 h. Furthermore, the conjugated siRNA exhibited 10-fold lower in dosage efficacy as compared to the commercially available siRNA [73]. A recent study by Choe et al. suggested to co-administrate L-type calcium channel blockers to further facilitate cellular internalization. As result, silencing of cp-asiRNA was potentiated without significant adverse effect [76]. Likewise, RXI-109, a cholesterol conjugated siRNA discovered by RXi Pharmaceuticals’, exhibited a reduction of CTGF during the course of wound healing followed by keloidectomy. This therapy was applicable for patients suffering from age-related macular degeneration with high risk of subretinal fibrosis (www.rxipharma.com/technology/rxi-109, accessed on 15 November 2021). Therefore, targeting CTGF with conjugated siRNA is a good direction for fibrotic disorders such as hypertrophic scars and keloids. Moreover, these ONs are anticipated to treat excess collagen from injury or after surgery which was conventionally treated with less effective silicon sheets with the application of pressure.

##### B. Fatty Acid Conjugates

Like cholesterol, fatty acid is an attractive entity for bioconjugation since it offered hydrophobicity customization and mimicked the uncanny composition of the phospholipid membrane. Currently, unbranched fatty acids were heavily delved such as the study conducted by Prakash et al. An array of fatty acid tethered to 16-mer-ASO through phosphodiester–linked hexaylamino spacer was synthesized. Two structure-activity relationship (SAR) studies were conducted and examined two revolving concepts: carbon length and degree of unsaturation. The first SAR involved with eight different fatty acids’ lengths (C10 to C22) conjugated to ASO revealed two findings: (1) protein binding property with chain length shorter than 16-C was lower than their counterparts showed in Table 3 and Table 4) Malat-1 expression was more significantly reduced by ASO with fatty.

Fatty acid chain longer than 12 in quadriceps while all remained similar in the heart [77]. Furthermore, the second SAR delved with 12 different unsaturated fatty acids. Protein binding to albumin, LDL, and HDL were slightly improved while there was no significant effect attributed to the double bond position. The activity of the representative unsaturated ASO displayed significant improvement compared to unconjugated ASO; however, there was no remarkable difference from their saturated counterparts. An interesting observation was none of the unsaturated moiety could outmatch palmitoyl’s silencing activity. Hence, palmitoyl conjugated ASO was selected as to be the most efficacious and subjected for elucidating muscle uptake mechanism in rodent model. Chappel et al. examined the consequential efficacy of palmitoyl-ASO after injection to endocytosis receptor knockdown mice (CAV1-/-, FcRN-/- and Alb-/-) [78]. In CAV knockdown mice, ED_50_ of palmitoyl ASO in quadriceps decreased by four-fold compared to wild type (9.7 µmol/kg versus 2.4 µmol/kg). In FCRN -/- mice, attenuation of palmitoyl ASO’s activity was observed compared to controlled groups with similar outcome (ED_50_ of 5.5 µmol/kg to 16 µmol/kg). A contrast was observed in Alb -/- mice with unchanged activity in quadriceps (0.73 µmol/kg in Alb -/- and 0.71 µmol/kg in controlled BL6). Thus, muscle uptake of palmitoyl-ASO was facilitated by caveolin-receptor-mediated endocytosis into endothelium cells once bound to albumin. Simultaneously, silencing FcRN could weaken the recycling of albumin into circulation thus impairing the albumin binding of ASO. However, Alb -/- mice contradicted the hypothesis, which would question if other proteins would be upregulated in compensation of drastic albumin reduction, and some would exist sufficient affinity for palmitoyl ASO binding.

Khvorova group compared the pharmacokinetic distribution property of diverse lipid moiety with emphasis on four fatty acids: myristic (Myr), docosahexaenoic (DHA), docosanoic (DCA), and eicosapentaenoic (EPA) acid. Length and degree of unsaturation constituted the hydrophobicity, which resulted in various in vivo distribution outcome. This study concluded with two premises: (1) more hydrophobic conjugates offered higher retention and (2) hydrophobicity instituted tissue accumulations [53]. Furthermore, shorter and less hydrophobic fatty acid such as myristic was synthesized and PK was analyzed as mono-, di-, or trimer. As discussed, the impact of hydrophobicity was profoundly shown in different behavior: (i) mono-lipid conjugates was quickly released with high kidney accumulation, (ii) di-lipid conjugates functioned as in-between showing preferential liver accumulation while flexibly distributed to other tissue (lung, heart, and fat), and (iii) tri-lipid conjugates resides at the injection site with no significant systemic exposure [79].

Table 5 summarized the pharmacokinetic parameter of three myristic variants. DCA-conjugated ON shared similar PK property as dimeric Myr and was able to silence the expression of myostatin (Mstn) in skeletal muscle after subcutaneous injection at 20 mg/kg dosage. Mstn, a growth factor expressed in skeletal muscles, negatively modulates muscle mass; hence, its inhibition was a potential therapeutic treatment against muscle wasting [80,81,82] or Duchenne muscular dystrophy (DMD) [83,84,85]. Interestingly, the toxicity profile of fatty acid conjugates was safer compared to cholesterol conjugates showing low activation of cytokine at high dose (100 mg/kg) [86].

Fatty acid would serve as an ideal conjugate to deliver therapeutic ON to muscle tissue. Currently, two ASO-splicing modulated therapy are approved by the FDA for muscle-related index such as eteplersen for Duchenne muscular dystrophy (DMD) [87] and nusinersen for spinal muscular atrophy [88]. Although eteplersen received such speedy approval with promising application, overall clinical efficacy [87,89] and renal toxicity from high dose [90] remained controversial. Thus, fatty acids can aspire to be a delivery platform to ameliorate both therapies for patients in need. Aside from muscular-related disorder, GRN163L (Imetelstat sodium) currently resides at phase III of clinical trials as a treatment for myelofibrosis as shown in Table 3 (fatty acid conjugate). It is a 13-mer phosphorothioate ASO with covalently attached palmitic acid at the 5′ terminal that exuded telomerase inhibitory activity. Observation of telomerase shortening was detected across multiple cancerous cells derived from glioblastoma [91], multiple myeloma [92], Barrett’s esophageal adenocarcinoma [93], breast [94], lung [95], and liver [96]. From in vivo delivery perspective, IC_50_ values were seven-fold higher [97], and efficacy increased up to 56% compared to naked counterpart after 24 h followed by intravenous injection (50 mg/kg) [98]. In follow-up studies, a group of researchers managed to explore the effects of long-term GRN163L exposure on the maintenance of telomeres and lifespan of 10 pancreatic cancer cells. They summarized the study with IC_50_ value was ranged from 50 nM to 200 nM, and suggested continuous exposure of GRN163L eventually led a complete loss of viability after several doubling times. Conversely, telomerase reactivation and elongation were observed in the absence of GRN163L. This observation reinforced that GRN163L could target the RNA template region of telomerase and proven to produced outstanding inhibitory effect. Overall, these outcomes demonstrated that the lifespan of pancreatic tumor cells can be shortened by continuous exposure and can be used in patients in the future [99]. Additionally, co-administration of GRN163L with trastuzumab revealed to produce synergistic effect, which GRN163L reversed the resistance of HER 2 + metastatic breast cancer against trastuzumab.

The clinical application of fatty acid conjugate is extended to ameliorate antibacterial resistance and antibiotic treatment as well. The attachment of ketal bis C15 and cyanine to 25-mer oligonucleotide at 5′ or 3′ terminal proved the efficient strategies in cell delivery. It decreased the minimum inhibitory concentration (MIC) of laboratory and clinical resistant strains to cephalosporin drug (i.e., ceftriaxone) by 25-fold than the naked equivalence. The decrease of beta-lactamase activity was dose-dependent and 5µM was found to be efficacious. Furthermore, 3′ lipid modification was less efficient than 5′. The 3’-attachment could propel the destabilization of heteroduplex structure of mRNA-LON, which enhanced steric hinderance to prevent RNase cleavage rather than uptaking into the bacteria [100]. 

##### C. Vitamin E (α-tocopherol)

Vitamin E is a group of fat-soluble compounds consisting of either tocopherol or tocotrienols. Naturally occurring α-tocopherol is an essential dietary supplement so it would be a safe and interesting selection for chemical bioconjugation. Additionally, its structure is composed of hydroxyl chromane and a hydrophobic saturated side chain that potentially enhances ON membrane permeability. Nishina et al. synthesized a 17-mer gapmer targeting murine hepatic ApoB. Structurally, it consisted of a parent 13-mer gapmer flanked by two wings of LNA with additional 4-mer modified RNA as the second wing directly linked to α-tocopherol via a phosphodiester bond. In vivo efficacy examination, tocopherol 17-mer ON showed −70% ApoB mRNA silencing capability after murine injection at 0.75 mg/kg [101]. The mRNA silencing potency was heavily dependent on dosage level (drastic reduction of ApoB expression as dose increased to 1.5 and 3 mg/kg) and prolonged duration of exposure (maximum response occurs from day 3 to 14). A followed-up pharmacokinetic study using Alexa Fluor-647 tagged tocopherol 17-mer ON at 5′-end revealed more than 3.5-fold higher of accumulation in the liver compared to non-conjugated parent, while tocopherol 17-mer ON also possessed higher serum content (10,000 µg/L) than naked parent ON (>1000 µg/L) at 5 min after 5 mg/kg dose of injection. The pharmacokinetic parameter of tocopherol 17-mer ON was summarized in Table 6. Interestingly, western blot analysis suggested cleavage of full-length tocopherol 17-mer ON into naked 13-mer unit once arrived at the liver which hypothesized the second wing tagged tocopherol acting as a delivery enhancer and release the main frame of 13-mer to initiate RNase-H cleavage mechanism toward targeted ApoB mRNA.

Another study conducted by Østergaard et al. comparing three different bioconjugates (cholesterol, tocopherol, and palmitate) ASO duplex targeting dystrophia myotonic protein kinase (DMPK), which caused myotic dystrophy (DM1) as the product of toxic repetition of nucleotide in the 3′- untranslated regions [102]. Structure of tocopherol-conjugated ON was illustrated in Table 3 (tocopherol conjugate). In vivo rat models, palmitate conjugated ASO responded with more improved silencing potency in skeletal muscle and heart compared to cholesterol and tocopherol after 10 mg/kg injection dose. However, in the monkey model, tocopherol conjugated-ASO came as more advantageous than the other two displaying a lower ED_50_ value of 7 mg/kg across three different DMPK expressed tissues (heart, quadriceps, and tibialis) [103]. Additionally, tocopherol moiety displayed tolerable high dose while cholesterol struggled with toxicity issues (in mice and unable to advance for primal testing). In plasma pharmacokinetics, tocopherol conjugates tended to co-elute with HDL and LDL, which displayed from size exclusion chromatography suggesting the essential of plasma protein binding was essential for receptor-mediated endocytosis. Benizri et al. disclosed additional pharmacokinetic data showing elevated liver accumulation after 6-h injection at a dose of 3 mg/kg (9–14 µg/g for tocopherol-ON versus 2–5 µg for naked-ON) [98]. However, tocopherol is remained understudied and required further preclinical investigation; thus, limited cases of human studies are often acquired.

##### D. Squalene

Squalene is a naturally occurring triterpene molecule that is frequently harvested from shark’s liver and some variety of vegetable oil. It is an important precursor for human cholesterol synthesis. As hydrophobic moiety squalene is also a candidate for ON conjugation that can couple either at 3′ or 5′ terminal of the sense strand. Thiol-maleimide or DBCO via click chemistry are usually generated, and squalene-ON can spontaneously form nanoparticles. Due to the amphiphilic nature of squalene, these nanoparticles could assemble in different shapes. Raouane et al. synthesized a 5′ squalene attached mRNA duplex employing a thiol-maleimide linker. This spherical nanoparticle was characterized by a drastic increase in lipophilicity while maintaining exceptional stability in serum media as a negative suspension (zeta potential= −26 mV). Cytotoxicity MTT assay in BHP-10-3 cell lines demonstrated > 95% cell viability at 50 nM maximal concentration of squalene-ON nanoparticle, while qRT-PCR depicted −80% RET/PTC1 silencing capability in vitro. Mice implanted with tumor were intravenously administered with a dose of 2.5 mg/Kg in vivo also demonstrated approximately 80% silencing of RET/PTC1 through qRT-PCR. Tumor biopsy showed significant shrinkage compared to controlled naked mRNA duplex after 15 days of the injection [43]. In another oncogenic targeting study, Masaad et al. investigated the silencing outcome of 5′ squalene attached ON against TMPRSS2-ERG fusion oncogene. This group employed Cu-free click chemistry to functionalize reactive DBCO group tethering to the spacer of siRNA duplex to azido squalene. The structure was shown in Table 3 (squalene conjugate). Nanoformulation of 5′ end was characterized to be temperature sensitive and degradable at 37 °C, while its structure was constricted to be spherical and quite anionic (zeta = −37 mV). This formulation was subjected to in vitro inhibitory efficacy test with VCap cells. Wherein, 50 nM of 5′-squalene nanoparticle showed a similar silencing effect (−50%) as naked siRNA transfected by lipofectamine after 3 different time points. Additionally, xenografted mice with VCap tumors showed significant size growth inhibition by −60%. siRNA treated mice were sacrificed and collected with excretory organs to analyze biodistribution by detecting radioactive ^32^P label. The majority of siRNA nanoparticles resided in either liver or kidney; however, it was interesting to see a significant accumulation directly at prostate tumors [42]. Hence, squalene conjugation was an exciting concept for ON’s design. Nevertheless, squalene harvesting can be controversial due to the revolving of endangering the shark population.

## 2. Conclusions and Future Outcome

Hydrophobic modifications such as cholesterol, fatty acid, α-tocopherol, and squalene still have room to mature compared to medicinal nanoformulation such as micelle, lipoplex, or, even, LNP. Of course, the primary goal of bioconjugation is elevating hydrophobic profile of ON-based therapy but a deeper quantitative understanding of structure related to delivery efficacy is still underappreciated. As mentioned in Biscan et al., the hydrophobicity profile of three distinctive lipid conjugates (dimeric Myr, cholesterol, and tocopherol succinate) appear to be similar as quantified via HPLC (measured in retention time); however, the biodistribution pathways are concluded to be diverse. Countless observation of multiple lipoid conjugates is accumulated at large in the secretory organ (liver) but detection at other tissues includes the spleen, kidney, and, even, skeletal muscle tissue will pave the way to develop novel delivery techniques to extrahepatic tissues [53]. Some fatty acids, such as docosanoic acid, had the ability to penetrate to skeletal muscle and, even, in the brain, which required direct spinal injection (unfavorable for human application) [37]. Aimed with current understanding, additional structural explorations would lead to better optimization for highly stable and selective modified ON; thus, current drawbacks in pharmacokinetic and biodistribution can be properly addressed. Moreover, there were still potential and unexplored lipophilic moiety both naturally occurred and artificial that can be examined to potentiate the delivery of ON-based therapy. More so, the profound pharmacokinetic, efficacy, and toxicity data from the previous conjugation can be utilized to develop a learning-based artificial intelligence to predict of other lipid species or even fabricate novel artificial structures in the quest of advancing ON-based therapy in the new height.

In the future, the hope of better delivery of ON therapy can reduce the need of a large dose to patients which can significantly cut down the cost of treatment. Currently, the patient-affordable cost for ON therapy is astronomical for individuals in need. Eteplirsen, the current treatment for DMD, was marketed in 2017 with the price of $300,000/patient a year; while nusinersen charges patients up to $750,000 for the first year following with $350,000 for consecutive years [104]. Such skyscraper cost of therapy can associate to denial of coverage from insurance companies. Even with approval, the insurance coverage may increase annually, which will devastate other members within the same insurance network. Therefore, the work of uncovering the most optimized delivery is not only limited to certain method but it is a combination effort of both bioconjugation and nanoparticle formulation.

## Figures and Tables

**Table 1:** List of FDA approved oligonucleotide drugs.

Name	Type	Modification	Mechanism	Indication/*Target*	FDA Approval
Fomivirsen	ASO	21 nt PS DNA	RNase H1	Cytomegalovirus retinitis/*CMV UL123*	Aug 1998
Pegaptanib	Aptamer	27 nt 2ʹ-F/2ʹ-OME PEGylated	Blocking binding	Neovascular age-related macular degeneration/*VEGF-165*	Dec 2004
Mipomersen	ASO Gapmer	20 nt PS 2ʹ-MOE	RNase H1	Homozygous familial Hypercholesterolemia/*APOB*	Jan 2013
Defibrotide	ssDNA and dsDNA	Mixture of PO	Non single sequence dependent based mechanism	hepatic veno-occlusive disease	Mar 2016
Nusinersen	Steric block ASO	18 nt PS 2′-MOE	Splicing, intron 7	Spinal muscular atrophy/*SMN2* *exon 7*	Dec 2016
Eteplirsen	Steric block ASO	30 nt PMO	Splicing, exon 51	Duchenne muscular dystrophy/*DMD* *exon 51*	Sep 2016
Milasen	ASO	22 nt PS 2′-MOE	Splicing	Batten disease/CLN7	Jan 2018
Patisiran	siRNA LNP formulation	19 + 2 nt 2′-OME	Ago2	Hereditary transthyretin amyloidosis, polyneuropathy-*TTR*	Aug 2018
Inotersen	Gapmer ASO	20 nt PS 2ʹ-MOE	RNase H1	hereditary transthyretin amyloidosis, polyneuropathy/*TTR*	Oct 2018
Givosiran	Dicer substrate siRNA	21/23 nt- GalNAc conjugate	Ago2	Acute hepatic porphyria *ALAS1*	Nov 2019
Golodirsen	Steric block ASO	25 nt PMO	Splicing, exon 53	Duchenne muscular dystrophy/*DMD exon 53*	Dec 2019
Viltolarsen	ASO	21 nt-PMO	Splicing	Duchenne muscular dystrophy/*DMD exon 53*	Aug 2020
Casimersen	ASO	22-PMO	Splicing	Duchenne muscular dystrophy/DMD Exon 45	Feb 2021
Inclisiran	siRNA	21/23 nt- GalNAc conjugate	Ago2	Hypercholesterolaemia/PCSKK9	Dec 2021

**Table 2:** Pharmacokinetic parameter of Chol-hsiRNA after intravenous injection adapted by Godinho et al.

Parameter (Units)	Chol-hsiRNA
k(min^−1^)	0.0013
*t*_1/2__α_(min)	515.8
*t*_1/2__β_ (min)	33.2
C_max_ (µg/mL)	753.4
AUC_0–48 h_ (µg/mL·min)	54,532.5
AUC_0-inf_ (µg/mL·min)	54,807.5
MRT_0-inf_ (min)	156.9
Vz (mL)	6.8
Cl (mL/min)	0.0091

**Table 3:** A selective example of ON conjugated with lipoid moieties in corresponding with each bioconjugates section.

Sequences	Spacer	Conjugates (X)	Source
Passenger 5’-CUUACCGACUGGAAGA-3’-**X** Guide 3’-CCGGACGGGAGCGCCGAAUGGCUGACCUUCU-5	**N/A**	Cholesterol	Hwang et al.
**X**-5’-TAGGGTTAGACAA-3’	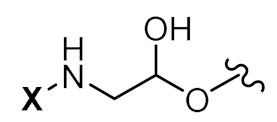	Palmitic acid (16C)	Herbert et al.
**X**-5’-TCAACAATAAATACCGAGG-3’	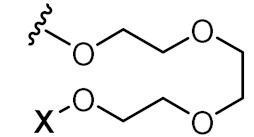	α-tocopherol	Østergaard et al.
Sense **X**-5’-GGAGGAACUCUCCUGAUGAAU-3’ Anti-sense 5’-UCAUCAGGAGAGUUCCUGCCG-3’	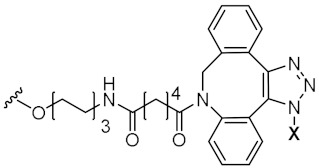	Squalene	Massaad-Massade et al.

Notation: red—2’MOE modification, green—cET modification, underline—PS backbone modification, and **X**—bioconjugates.

**Table 4:** Adapted protein binding data from Prakash et al. displayed the trends depending on carbon lengths.

Sequence	Conjugates (X = 5’-end)	Albumin Ki (µM)	LDL Ki (µM)	HDL Ki (µM)
GCATTCTAATAGCAGC	None	24.00	N/A	N/A
X- GCATTCTAATAGCAGC	C8 (Octanoyl)	2.20	11.80	5.80
X- GCATTCTAATAGCAGC	C10 (Decanoyl)	4.99	3.20	1.70
X- GCATTCTAATAGCAGC	C12 (Dodecanoyl)	3.22	1.30	0.75
X- GCATTCTAATAGCAGC	C14 (Myristoyl)	1.97	0.36	0.79
X- GCATTCTAATAGCAGC	C16 (Palmitoyl)	0.92	0.13	0.79
X- GCATTCTAATAGCAGC	C18 (Stearoyl)	0.85	0.17	0.66
X- GCATTCTAATAGCAGC	C20 (Eicosanoyl)	0.91	0.22	1.26
X- GCATTCTAATAGCAGC	C22 (Docosanoyl)	0.97	0.31	1.30

**Table 5:** Pharmacokinetic parameters of three myristic variants after 7 days period injection adapted from Biscan et al.

Parameter (Units)	Monomeric Myr	Dimeric Myr	Trimeric Myr
k_abs_ (min^−1^)	0.0562	0.0213	0.0341
t_1/2 abs_ (min)	12.3	32.5	20.3
k_β_ (min^−1^)	0.0218	0.0050	0.0015
t_1/2__β_ (min)	54.1	139.0	465.3
T_max_ (min)	60	120	120
C_max_ (µg/mL)	21.4	6.1	0.9
AUC (µg/mL*min)	3768.1	3511.1	984.6
MRT (min)	644.7	1534.5	2009.6

**Table 6:** Pharmacokinetic parameter of Toc-17-mer ASO.

Parameter	Toc-17-mer ASO
AUC (∞) (ug/mL·min)	379 ± 14
CLtot (mL/min/g)	0.0079 ± 0.0005
MRT (min)	32 ± 1
Vdss (mL/g)	0.252 ± 0.023
K_α_ (min^−1^)	0.0571 ± 0.0041
K_β_ (min^−1^)	0.00272 ± 0.00137

AUC—area under the serum concentration time curve, CLtot—total body clearance, MRT—mean residence rate constant, K_α_—initial elimination rate constant, K_β_—terminal elimination rate constant, and Vdss—steady-state volume of distribution.
